# The Bovhyaluronidase Azoximer (Longidaza^®^) Disrupts *Candida albicans* and *Candida albicans*-Bacterial Mixed Biofilms and Increases the Efficacy of Antifungals

**DOI:** 10.3390/medicina58121710

**Published:** 2022-11-23

**Authors:** Alina Gatina, Elena Trizna, Alena Kolesnikova, Diana Baidamshina, Anna Gorshkova, Valentin Drucker, Mikhail Bogachev, Airat Kayumov

**Affiliations:** 1 Institute of Fundamental Medicine and Biology, Kazan Federal University, 420008 Kazan, Russia; 2 Limnological Institute of the Siberian Branch of the Russian Academy of Sciences, 664000 Irkutsk, Russia; 3 Biomedical Engineering Research Centre, St. Petersburg Electrotechnical University, 197022 St. Petersburg, Russia

**Keywords:** *Candida albicans*, biofilms, enzymatic destruction of the biofilm, bovhyaluronidase azoximer (Longidaza^®^)

## Abstract

*Background and Objectives: Candida albicans* causes various diseases ranging from superficial mycoses to life-threatening systemic infections often associated with biofilm formation, including mixed fungal–bacterial consortia. The biofilm matrix protects cells, making *Candida* extremely resistant to treatment. Here, we show that the bovhyaluronidase azoximer (Longidaza^®^) in vitro destroys the biofilm formed by either *C. albicans* alone or mixed with bacteria, this way decreasing the concentrations of antimicrobials required for the pathogen’s eradication. *Materials and Methods:* Bovhyaluronidase azoximer, Longidaza® was obtained from NPO Petrovax Pharm Ltd., Moscow, Russia as lyophilized powder. The antifungal activity was assessed by microdilution assay and CFUs counting. Antibiofilm activity was evaluated via biofilms staining and scanning electron microscopy. *Results:* Thus, treatment with Longidaza^®^ reduced the biofilm biomass of nine *C. albicans* clinical isolates by 30–60%, while mixed biofilms of *C. albicans* with various bacteria were destroyed by 30–40%. Furthermore, the concentration of fluconazole required to achieve a similar reduction of the residual respiratory activity of detached cell clumps of four *C. albicans* isolates has been reduced four-fold when combined with Longidaza^®^. While in the biofilm, two of four isolates became significantly more susceptible to fluconazole in combination with Longidaza^®^. *Conclusion:* Taken together, our data indicate that Longidaza^®^ is capable of suppression of tissues and artificial surfaces biofouling by *C. albicans* biofilms, as well as facilitating drug penetration into the cell clumps, this way decreasing the effective MIC of antifungals.

## 1. Introduction

*Candida albicans* is the most prevalent fungi of the human microbiota. In healthy humans, it asymptomatically colonizes various niches, like the oral cavity, gastrointestinal and reproductive tracts, as well as the skin surface [1]. In immunocompromised patients, *Candida* causes various diseases, from mucosa mycoses to life-threatening systemic bloodstream infections [2]. The majority of mucosa candidiasis cases are associated with biofilm formation [3]. In the biofilm, cells are embedded into a self-produced matrix of various organic substances, like polysaccharides, proteins, lipids, and nucleotides [4]. The biofilm matrix provides adhesion and mechanical stability of the cell consortia, acting as an extracellular digestive system and providing a mechanical diffusional barrier for toxic compounds [5,6,7,8]. Thus, the biofilm protects cells from the immune system and antifungals, making *Candida* biofilms extremely resistant to treatment [9,10].

In immunocompromised patients, *C. albicans* commonly exists in the form of mixed bacterial–fungal biofilms consisting of two-, three-, or more species. In mixed consortia, fungal–bacterial interactions result in drastic alterations in drug susceptibility and pathogenicity of both organisms [2,11,12,13]. Regarding bloodstream infections, *Staphylococcus* species are the most frequent counterpart in consortia with *C. albicans*, while *Pseudomonas aeruginosa* is commonly co-isolated with *C. albicans* from skin and lung infections [14,15,16]. Under anaerobic conditions, *C. albicans* forms dual-species biofilms with *Bacteroides fragilis*, *Clostridium perfringens*, *Escherichia coli*, *Klebsiella pneumoniae*, or *Enterococcus faecalis* [17]. Mixed fungal–bacterial biofilms often differ significantly from their single-species counterparts, challenging the development of their efficient eradication options [18].

To date, several strategies for targeting mono- and polymicrobial biofilms have been proposed, including antimicrobial peptides, quorum-quenching compounds, and universal antiseptics with dual activity, etc. [19,20,21]. Among them, enzymatic hydrolysis of the biofilm matrix appears to be a promising approach because of the low toxicity and biodegradability of the enzymes, as well as absence of resistance development risks. Thus, the destruction of the matrix facilitates the penetration of antimicrobials into the cells and decreases the adhesion [22,23]. For example, dispersin B, the glycoside hydrolase produced by the periodontal pathogen *Aggregatibacter actinomycetemcomitans*, led to the destruction of *S. epidermidis* biofilms, resulting in increased sensitivity of bacteria to antimicrobials [24,25]. Various proteases like subtilisin A [26], Ficin [27,28], Papain [29], chymotrypsin [30,31], and others were reported as enhancers of antimicrobials against biofilm-embedded bacteria. Additionally, DNAse was reported to increase the efficacy of antimicrobial photodynamic therapy on *C. albicans* biofilms [32]. In another study, synergistic enzyme complex Lyticase was shown to disrupt biofilm formation by *C. albicans* [33]. Nevertheless, lack of data on the enzymatic treatment of *Candida* biofilms available to date appears to be a limiting factor for the understanding of its efficacy. Here, we show the effect of bovhyaluronidase fused with a copolymer of 1,4-ethylenepiperazine N-oxide and (N-carboxymethyl)-1,4-ethylenepiperazinium bromide (bovhyaluronidaze azoximer, Longidaza^®^) [34] on *C. albicans* mixed biofilms. The presence of azoximer provides anti-inflammatory activity of the drug, significantly prolongs the half-life of the enzyme’s activity, and allows broad modes of application, like vaginal suppositories and injections [34], thereby resolving objective difficulties of enzymes application, like low stability and few possible application methods (see [35,36,37] and references therein). In several clinical studies, the effect of bovhyaluronidaze azoximer (Longidaza^®^) on the microbiota of the urogenital tract has been reported [38,39,40,41]. Here, we show that Longidaza^®^ efficiently destroys *C. albicans* and *C. albicans*-bacterial mixed biofilms in vitro and increases the efficacy of antimicrobials, thus appearing as a beneficial tool to improve the treatment of *Candida* biofilm-associated infections.

## 2. Materials and Methods

### 2.1. Chemicals

Bovhyaluronidase azoximer, Longidaza^®^ was obtained from NPO Petrovax Pharm Ltd., Moscow, Russia as lyophilized powder in vials by 3000 International Units (IU) per vial. Cellulase and Ficin were used as the control and purchased from Sigma, St. Louis, MO, USA (C22178 and F4165, respectively). Other chemicals were reagent grade and purchased from Sigma, St. Louis, MO, USA.

### 2.2. Strains and Growth Conditions

*Candida albicans* clinical isolates were obtained from Kazan Institute of Microbiology and Epidemiology (Kazan, Russia). Their source and susceptibility to fluconazole are listed in Table 1. Fungi were identified by using the AuxaColor 2 Colorimetric sugar-assimilation yeast-identification kit (Bio-Rad). The isolate *C. albicans* 4940 was used in all extended experiments as a fluconazole and terbinafine sensitive strain. All strains were stored as a 50% glycerol stock at −80 °C and grown in BM broth (Basal medium, glucose 10 g, peptone 7 g, MgSO_4_ × 7 H_2_O 2.0 g, and CaCl_2_ × 2 H_2_O 0.05 g in 1.0 L tap water) [42]. To obtain mature biofilms, *C. albicans* cells were grown in BM broth in culture plates (tissue culture treated) under static conditions for 48 h at 37 °C. 

*Staphylococcus aureus* ATCC^®^ 29213™, *Escherichia coli* MG1655, *Pseudomonas aeruginosa* ATCC^®^ 27853™, and *Klebsiella pneumoniae* (clinical isolate) were chosen as the most frequent counterparts in *Candida albicans*–bacterial mixed consortia. Clinical isolate of *K. pneumoniae* was obtained from the Kazan Institute of Epidemiology and Microbiology (Kazan, Russia). Bacterial strains were stored as a 50% glycerol stock at −80 °C, while maintained on the LB medium (Luria-Bertani broth, Miller, Sigma-Aldrich). Bacteria were inoculated with *C. albicans* in BM broth and grown for 48 h without shaking at 37 °C. 

### 2.3. Minimum Inhibitory Concentrations (MICs)

Minimum inhibitory concentrations (MICs) of antifungals were determined using the broth microdilution method in 96-well plates (Eppendorf) in BM broth as recommended in the protocol CLSI M27-A3. *C. albicans* was grown overnight and diluted with BM broth until optical density of 0.5 at 600 nm to obtain the working solution. Then, 2-fold serial dilutions of antifungals in concentrations from 1 to 1024 μg/mL were prepared in BM broth and seeded with fungi (1% *v*/*v* of working solution) with subsequent incubation at 37 °C for 24 h. The MIC was defined as the lowest concentration of the compound at which no visible growth could be seen.

### 2.4. Biofilms Staining and Quantification 

To obtain biofilms, bacteria (2–9 × 10^6^ CFU/mL) and fungi (1–5 × 10^5^ CFU/mL) were grown under static conditions for 48 h in BM broth in 24-well TC-treated polystyrol plates (1 mL per well). Then, the broth was exchanged with a fresh one supplemented with Longidaza^®^ at concentrations as indicated. Ficin and Cellulase were used as reference enzymes able to disrupt bacterial biofilms. After 24 h incubation, the plates were subjected to crystal violet staining [43] and Congo Red depletion assay [44]. To quantify the amount of extracellular matrix of biofilms using Congo Red, a Congo Red solution in LB with a final concentration of 80 µg/mL was added to mature biofilms. After, biofilms were mechanically peeled off and incubated with dye for 90 min at 37 °C. Next, the plates were centrifuged for 5 min at 4400 rpm, the supernatant was transferred to 96-well plates, and the uncoated dye was measured using a Tecan infinite 200 Pro microplate reader (Switzerland) at 490 nm. To quantify the amounts of proteins and polysaccharides in the biofilm matrix, biofilms were washed once with 1×PBS and stained with Sypro Orange (ready to use ×1000 solution), ConA-TMR (500 µg/mL), or Calcofluor White M2R (CFW, 1 mg/mL). After 15 min incubation at 37 °C, wells were washed with PBS, filled with 100 µL PBS, and the fluorescence was measured on a microplate reader Tecaninfinite 200 Pro (Switzerland, Männedorf) at the following wavelengths: 470/570 nm for Sypro Orange (proteins), 552/578 nm for ConA-TMR (α-polysaccharides), 254/432 nm for Calcofluor White (β-polysaccharides). The amounts of proteins and polysaccharides were expressed in relative units calculated as fluorescence units normalized by total biofilm biomass assessed in the CV-stain. 

### 2.5. The Anti-Biofilm Activity

To analyze the effect of enzymes on cells in biofilms, the viability of bacteria was assessed by drop-plate analysis as described in Baidamshina et al. [27]. To do this, enzymes were added to mature 48-h biofilms in the required concentrations, followed by incubation for 24 h. After that, the wells were washed with a 0.9% NaCl, the biofilms were torn off mechanically, and after a series of tenfold dilutions, they were sown on an agar nutrient medium. After 24 h, the CFU were counted. 

To assess the effect of Longidaza^®^ on activity of antifungals against biofilm-embedded *C. albicans*, the 48 h-old *C. albicans* biofilms were established in 96-well flat-bottom polystyrene microplates by inoculation of the overnight culture in BM broth. Then, the plates were washed with sterile 0.9% NaCl and 200 μL of the fresh BM broth, containing antifungal in concentrations as indicated, were added following incubation for 24 h. In experimental wells, the broth contained an additional 3000 IU of Longidaza^®^. The viability of detached and biofilm-embedded cells was evaluated by MTT-assay [45]. 

### 2.6. Scanning Electron Microscopy 

The structure of mixed fungal–bacterial biofilms after treatment with Longidaza^®^ was assessed with scanning electron microscopy. The biofilms were established by seeding fungal–bacterial suspension in BM broth in 34 mm plastic adhesive Petri dishes (TC-treated, Eppendorf, 2 mL per plate) followed with 48 h growth at 37 °C under static conditions. Mature biofilms were washed with sterile PBS, filled with fresh BM broth containing 750 IU of Longidaza^®^ and incubation was followed for the next 24 h. Then, plates were washed 3 times with water and fixed with glutaraldehyde (1% water solution) for 24 h. After subsequent washing with deionized water, the plates were dried 12 h at 55 °C and coated in vacuum with gold on SCD 004 (Balzers AG, Balzers, Liechtenstein). SEM was performed with the Quanta 200 microscope (FEL Company, Skokie, IL, USA) at 29 kV at the Ultramicroanalysis Research Center at the Limnological Institute of the Siberian Branch of the Russian Academy of Sciences, Irkutsk.

### 2.7. Statistical Analysis

Experiments were carried out in biological triplicates (i.e., newly prepared cultures and medium) with three independent repeats in each one. The statistical significance of results was assessed using the Kruskal–Wallis statistical test with significance threshold at *p* < 0.05 in Prism 6 (GraphPad Software Inc. San Diego, CA, USA).

## 3. Results 

### 3.1. The Effect of Bovhyaluronidaze Azoximer (Longidaza^®^) on C. albicans Biofilms

**Figure 1:**
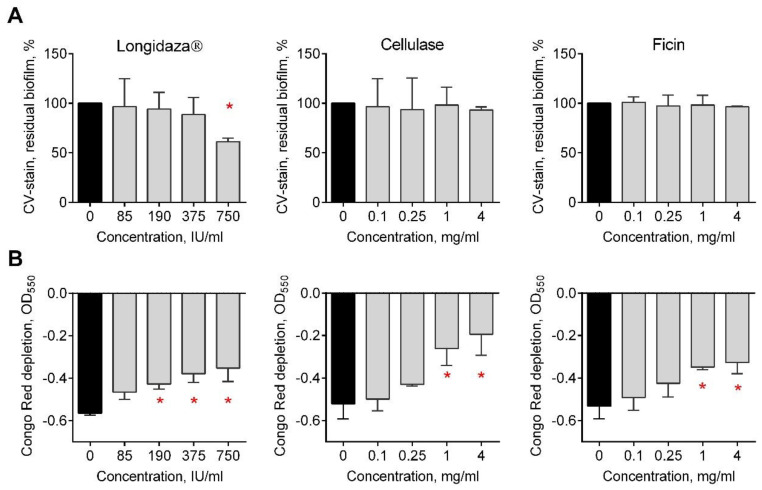
The effect of Longidaza^®^, Cellulase, and Ficin on *C. albicans* K4940 biofilms in vitro. Forty-eight-h-old *C. albicans* biofilms were washed and incubated 24 h in fresh BM broth supplemented with either 85−750 IU of Longidaza^®^, 0.1−4 mg/mL of Cellulase from *Aspergillus niger*, or Ficin as indicated. Biofilms were quantified with either (**A**) crystal violet staining or (**B**) Congo Red depletion assay. Asterisks denote significant difference with untreated samples (* *p* < 0.05).

In several clinical studies, a possible effect of bovhyaluronidaze azoximer (Longidaza^®^) on the microbial biofilms in the urogenital tract has been reported. Therefore, we investigated whether Longidaza^®^ is capable of destructing in vitro the biofilms of *C. albicans*. For that, 48-h-old biofilm of *C. albicans* clinical isolate was prepared and treated for 24 h with Longidaza^®^ at various concentrations. Next, Cellulase from *Aspergillus niger* and protease Ficin, for which the biofilm-destruction activity has been reported previously [27,46], were used as relevant controls. As shown in Figure 1A, treatment with 750 IU of Longidaza^®^ led to the reduction of the biofilm biomass by 30%. To confirm the destruction of the biofilm matrix, the Congo Red depletion assay was performed. Significant reduction of the dye adsorption with biofilms treated with Longidaza^®^ was observed in a dose-dependent manner, suggesting the destruction of the biofilm matrix (Figure 1B). While no reduction of biofilm biomass treated with either Cellulase or Ficin could be detected in crystal violet stain, in the Congo Red assay, a dose-dependent decrease of the biofilm matrix was observed. 

**Figure 2:**
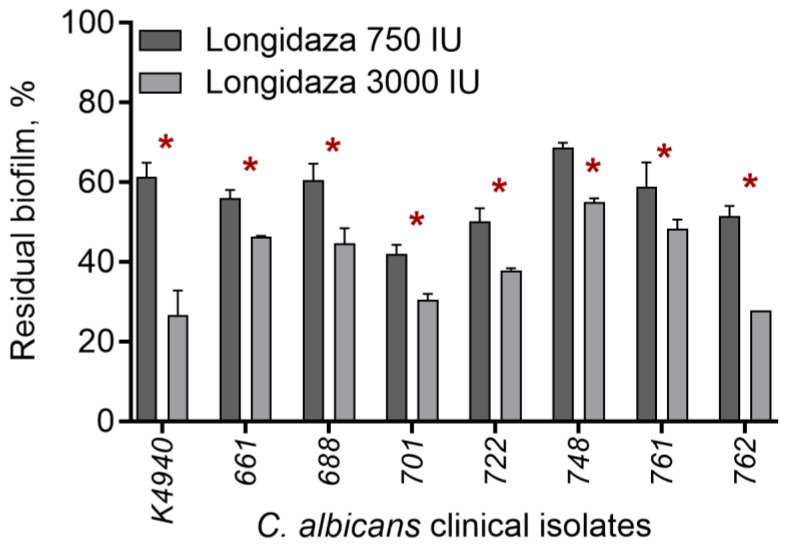
The in vitro destruction of biofilms formed by *C. albicans* clinical isolates. Forty-eight-h-old *C. albicans* biofilms were washed and incubated 24 h in fresh BM broth supplemented with 750 IU or 3000 IU of Longidaza^®^. Biofilms were quantified with crystal violet staining. Asterisks denote significant difference with untreated samples (* *p* < 0.05).

Since the biofilm rigidity and matrix compositions vary significantly for different *C. albicans* strains, we tested the effect of Longidaza^®^ on 7 additional clinical isolates. As could be seen from Figure 2A, after treatment with 750 ME of the enzyme, the residual biofilm of isolates varied in the range of 40–70%. The increase in the Longidaza^®^ dose to 3000 IU led to deeper biofilm destruction of all clinical isolates, and the residual biofilm decreased to 20–55% of initial values (Figure 2B), suggesting Longidaza^®^ as a promising tool for the destruction of *C. albicans* biofilms. To reveal the reason of various efficacy of the enzyme on biofilms formed by different isolates, the quantity of proteins, α-, and β-polysaccharides were assessed by differential fluorescent staining (Table 2). As could be seen from the Table 2, the relative amount of proteins and polysaccharides differs drastically between isolates. In turn, a significant correlation was observed between the relative content of α- and β-polysaccharides and proteins and the biofilm reduction after treatment with Longidaza^®^ (CV stain). Thus, the *C. albicans* 748 biofilm was less effectively destroyed, in which the maximum content of all the studied components was noted.

### 3.2. The Effect of Bovhyaluronidaze Azoximer (Longidaza^®^) on C. albicans–Bacterial Mixed Biofilms

**Figure 3:**
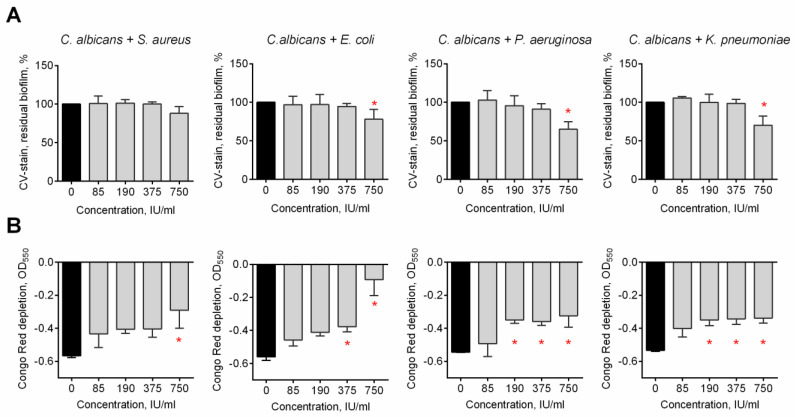
The effect of Longidaza^®^ on fungal–bacterial biofilms. forty-eight-h-old fungal–bacterial biofilms were washed and incubated 24 h in fresh BM broth supplemented with 85−750 IU of Longidaza^®^ as indicated. Biofilms were quantified with either (**A**) crystal violet staining or (**B**) Congo Red depletion assay. Asterisks denote significant difference with untreated samples (* *p* < 0.05).

*C. albicans* can form fungal–bacterial mixed biofilms which are generally known to exhibit higher resistance to various treatment options [1,47,48,49]. Therefore, mixed biofilms of *S. aureus*, *E. coli*, *P. aeruginosa*, and *K. pneumoniae* with *C. albicans* were prepared and treated with Longidaza^®^. Crystal violet staining revealed more pronounced reduction of the biofilm biomass for the *C. albicans*–*P. aeruginosa* consortium, with 50% of residual biofilm at the highest concentration of the enzyme (Figure 3A). For all other dual-species biofilms, a significant biomass reduction could be detected only at 750 IU of Longidaza^®^. Nevertheless, the Congo Red assay data clearly demonstrated that treatment with Longidaza^®^ reduces the matrix biomass of all mixed cultures in a dose-dependent manner (see Figure 3B).

**Figure 4:**
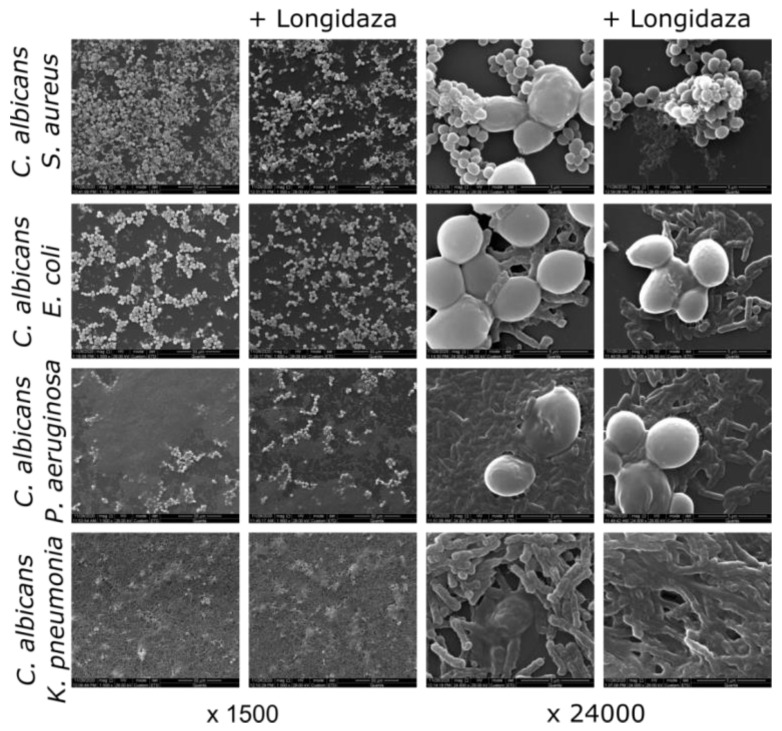
The effect of Longidaza^®^ on fungal–bacterial biofilms. Forty-eight-h-old fungal–bacterial biofilms were washed and incubated 24 h in fresh BM broth supplemented with 750 IU of Longidaza^®^ as indicated and analyzed with scanning electron microscopy.

**Figure 5:**
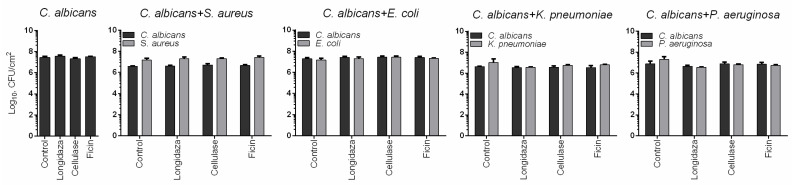
The effect of Longidaza^®^ on CFUs count in *C. albicans* and fungal–bacterial biofilms. Forty-eight-h-old biofilms were washed and incubated 24 h in fresh BM broth supplemented with 750 IU of Longidaza^®^ as indicated. After 24 h incubation, the biofilms were washed twice with sterile 0.9% NaCl. The adherent cells were scratched, resuspended, and CFUs were counted.

The structures of non-treated and Longidaza^®^-treated biofilms were analyzed with scanning electron microscopy (Figure 4). A visible decrease of the biofilm treated with Longidaza was observed for *C. albicans*–*S. aureus* and *C. albicans–P. aeruginosa* biofilms, although no decrease in the amount of viable bacterial and fungal cells in the consortia has been confirmed by the CFUs count (Figure 5).

### 3.3. The effect of Longidaza^®^ on the Susceptibility of Biofilms-Embedded C. albicans to Antifungals

Being embedded into the biofilm matrix, *C. albicans* becomes largely inaccessible to conventional antifungals. We tested whether the incubation with Longidaza^®^ would increase the efficiency of antifungals against adherent fungal cells and swimming cell clumps dispersed from the biofilm. For that, 48-h-old biofilms of four *C. albicans* clinical isolates (4940, 661, 688, 701) were prepared and fluconazole at different concentrations was added either alone or in combination with Longidaza^®^ (in concentration of 3000 IU) to the established biofilms. After 24 h incubation, the viability of both detached and biofilm-embedded cells was assessed in MTT-assay.

**Figure 6:**
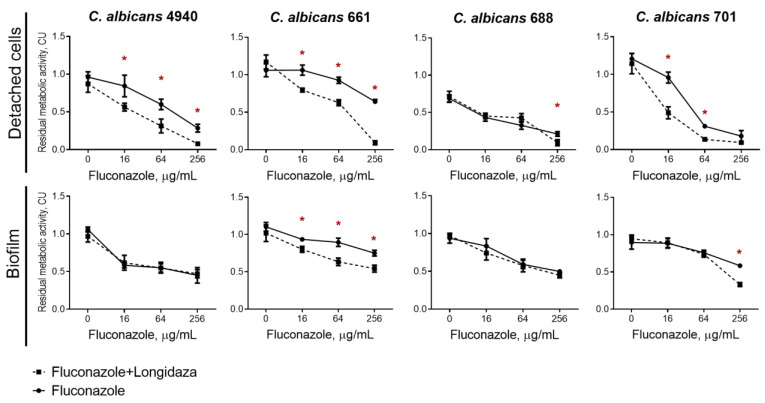
The effect of Longidaza^®^ on the susceptibility of detached and biofilm-embedded *C. albicans* cells to fluconazole. Longidaza^®^ was added to 48-h-old biofilms to a final concentration of 3000 IU/mL. Fluconazole was added up to final concentrations of 16−256 µg/mL. After 24 h incubation, the biofilms were washed twice with sterile 0.9% NaCl. The viability of cells was analyzed with an MTT-assay. The asterisks (*) denote a statistically significant difference of the residual respiratory activity in the untreated wells (solely antimicrobials) and wells with the combined treatment (*p* < 0.05).

Longidaza^®^ itself did not affect the viability of *C. albicans* (Figure 6, point 0). Treatment with even 256 µg/mL of fluconazole did not lead to full death of cells (Figure 6), indicating inefficiency of solely antifungal treatment. In marked contrast, the maximal concentration of fluconazole in combination with Longidaza^®^ led to the complete death of detached cell clumps (Figure 6 upper row). At the same time, the combined use of the enzyme with antifungal increased the effectiveness of the latter by four times against detached cells clumps in three out of four studied isolates. The combined use of fluconazole with the enzyme was less effective against cells in the biofilm—only two strains out of four tested became statistically more susceptible to fluconazole in the presence of the Longidaza^®^. Nevertheless, to achieve a similar effect on *C. albicans* 661 biofilm, a 16-fold lower concentration of the antifungal drug was required in combined use compared to monotherapy. An increase in the effectiveness of fluconazole against cells in the biofilm of *C. albicans* 701 has also been shown, although at the maximum concentration of the antifungal (Figure 6 lower row).

## 4. Discussion

*Candida albicans* asymptomatically colonizes various niches in human body, like the oral cavity, gastrointestinal and reproductive tracts, causes various diseases In immunocompromised patients [2]. The majority of mucosa candidiasis cases are associated with biofilm formation [3], where cells are embedded into a self-produced matrix and thus protected from toxic compounds, the immune system and antifungals [5,6,7,8]. 

In several clinical studies, a possible effect of bovhyaluronidaze azoximer on the microbial biofilms in the urogenital tract has been reported. Our data show that Longidaza^®^ is capable of destructing in vitro the biofilms of *C. albicans* by 30% after the treatment with 750 IU of the enzyme in a dose-dependent manner, while no reduction of biofilm biomass treated with either Cellulase or Ficin could be detected (Figure 1). On a clinical isolates, the residual biofilm of isolates varied in the range of 40–70%, apparently, since the relative amount of proteins and polysaccharides differs drastically between isolates (Table 2). This fact explains a significant correlation observed between the relative content of α- and β-polysaccharides and proteins and the biofilm reduction after treatment with Longidaza^®^ (CV stain). Thus, the *C. albicans* 748 biofilm was less effectively destroyed, in which the maximum content of all the studied components was noted.

*C. albicans* can form fungal–bacterial mixed biofilms which are generally known to exhibit higher resistance to various treatment options. Among the most frequent bacteria-forming consortia with *C. albicans* at the urogenital infection, *S. aureus*, *E. coli*, *P. aeruginosa*, and *K. pneumoniae* are mentioned in various reports [1,47,48,49]. The pronounced reduction of the biofilm biomass has been observed for the *C. albicans*–*P. aeruginosa* consortium, with 50% of residual biofilm at the highest concentration of the enzyme (Figure 3A), apparently, because of the lowest ratio of proteins and polysaccharides in the biofilm. For all other dual-species biofilms, a significant biomass reduction could be detected only at the highest concentration of Longidaza^®^. The observed data were confirmed with scanning electron microscopy (Figure 4). A visible decrease of the biofilm treated with Longidaza was observed for *C. albicans*–*S. aureus* and *C. albicans–P. aeruginosa* biofilms, although no decrease in the amount of viable bacterial and fungal cells in the consortia has been confirmed by the CFUs count (Figure 5). Consequently, the enzyme leads to hydrolysis of the extracellular matrix, but does not lead to cell death in the biofilm.

Being embedded into the biofilm matrix, *C. albicans* becomes largely inaccessible to conventional antifungals. While the Longidaza^®^ itself did not affect the viability of *C. albicans* (Figure 6, point 0), the fluconazole in combination with Longidaza^®^ led to the complete death of detached cell clumps (Figure 6 upper row). At the same time, the combined use of the enzyme with antifungal increased the effectiveness of the latter by four times against detached cells clumps in three out of four studied isolates. The combined use of fluconazole with the enzyme was less effective against cells in the biofilm—only two strains out of four tested became statistically more susceptible to fluconazole in the presence of the Longidaza^®^. Nevertheless, to achieve a similar effect on *C. albicans* 661 biofilm, a 16-fold lower concentration of the antifungal drug was required in combined use compared to monotherapy. Probably, the lack of effect on other strains is due to the difference in the composition of the extracellular matrix of the biofilm and, as a consequence, the different permeability for antimicrobials, regardless of the presence of the enzyme in the medium (Table 2). While many enzymes were reported to be efficient in destruction of bacterial biofilms [27,28,29,50,51,52,53,54,55,56], relatively low works show the enzymatic destruction of fungal and fungal–bacterial biofilms [33,57,58]. Taken together, our data and literature data allow assuming that treatment of fungal biofilms remains challenging in modern infection medicine and efficient tools for targeting fungal and fungal–bacterial biofilms are required to be developed.

## 5. Conclusions

Taken together, our data demonstrate that Longidaza^®^ is capable of destruction of the biofilm formed by *C. albicans*, including *C. albicans*–bacterial consortia. This provides a combined effect, including reduction of the biofouling of tissues and artificial surfaces, as well as facilitating the drug penetration into the biofilm matrix, this way also reducing the effective MIC of antifungals. Thus, a combination of antifungal with Longidaza^®^ treatment could significantly increase the efficiency of biofilm-associated fungal and fungal–bacterial infections treatment.

## Figures and Tables

**Table 1:** Source of *C. albicans* clinical isolates and their susceptibility to fluconazole.

Isolate	Source	MIC, µg/mL
*C. albicans K4940*	Buccal swab	64
*C. albicans 661*	Pharynx, mucosa of the tongue	1024
*C. albicans 688*	Mucosa of the pharynx	1024
*C. albicans 701*	Mucosa of tonsils	1024
*C. albicans 722*	Mucosa of the pharynx	1024
*C. albicans 748*	Mucosa of the pharynx	64
*C. albicans 761*	Vaginal swab, cervical canal	1024
*C. albicans 762*	Mucosa of the urethra	1024

**Table 2:** Comparative assessment of the total biomass of biofilms and the relative content of polysaccharides and proteins in the matrix of biofilms of *C. albicans* isolates before and after treatment with Longidaza^®^.

Isolate	Total Biomass of the Biofilm, OD_570_	Residual Biomass of the Biofilm, OD_490_	α-Polysacch, Relative Units	β-Polysacch, Relative UNits	Proteins, Relative Units
*C. albicans K4940*	0.11	0.07	22.80	22.80	7.01
*C. albicans 661*	0.12	0.07	19.13	16.64	11.65
*C. albicans 688*	0.10	0.07	37.48	40.37	11.53
*C. albicans 701*	0.15	0.06	17.90	9.94	7.95
*C. albicans 722*	0.13	0.06	19.36	18.58	4.65
*C. albicans 748*	0.09	0.06	38.35	41.84	27.89
*C. albicans 761*	0.11	0.07	25.99	23.20	21.35
*C. albicans 762*	0.13	0.07	12.38	13.15	6.19

## Data Availability

The data presented in this study are available on request from the corresponding author.
